# Associations between dietary and blood inflammatory indices and their effects on cognitive function in elderly Americans

**DOI:** 10.3389/fnins.2023.1117056

**Published:** 2023-02-21

**Authors:** Wanyue Li, Shuna Li, Yaru Shang, Weisheng Zhuang, Guoqiang Yan, Zhuoming Chen, Jun Lyu

**Affiliations:** ^1^Department of Rehabilitation, The First Affiliated Hospital of Jinan University, Guangzhou, Guangdong, China; ^2^Department of Clinical Research, The First Affiliated Hospital of Jinan University, Guangzhou, Guangdong, China; ^3^Department of Rehabilitation, Henan Provincial People’s Hospital, People’s Hospital of Zhengzhou University, Zhengzhou, Henan, China; ^4^Guangdong Provincial Key Laboratory of Traditional Chinese Medicine Informatization, Guangzhou, Guangdong, China

**Keywords:** cognitive function, DII, blood inflammation indicators, NHANES, regression analysis

## Abstract

**Objective:**

To determine the correlations between dietary and blood inflammation indices in elderly Americans and their effects on cognitive function.

**Methods:**

This research extracted data from the 2011–2014 National Health and Nutrition Examination Survey for 2,479 patients who were ≥60 years old. Cognitive function was assessed as a composite cognitive function score (Z-score) calculated from the results of the Consortium to Establish a Registry for Alzheimer’s Disease Word Learning and Delayed Recall tests, the Animal Fluency test, and the Digit Symbol Substitution Test. We used a dietary inflammatory index (DII) calculated from 28 food components to represent the dietary inflammation profile. Blood inflammation indicators included the white blood cell count (WBC), neutrophil count (NE), lymphocyte count (Lym), neutrophil–lymphocyte ratio (NLR), platelet–lymphocyte ratio (PLR), neutrophil–albumin ratio (NAR), systemic immune-inflammation index [SII, calculated as (peripheral platelet count) × NE/Lym], and systemic inflammatory response index [SIRI, calculated as (monocyte count) × NE/Lym]. WBC, NE, Lym, NLR, PLR, NAR, SII, SIRI, and DII were initially treated as continuous variables. For logistic regression, WBC, NE, Lym, NLR, PLR, NAR, SII, and SIRI were divided into quartile groups, and DII was divided into tertile groups.

**Results:**

After adjusting for covariates, WBC, NE, NLR, NAR, SII, SIRI, and DII scores were markedly higher in the cognitively impaired group than in the normal group (*p* < 0.05). DII was negatively correlated with the Z-score when combined with WBC, NE, and NAR (*p* < 0.05). After adjusting for all covariates, DII was positively correlated with SII in people with cognitive impairment (*p* < 0.05). Higher DII with NLR, NAR, SII, and SIRI all increased the risk of cognitive impairment (*p* < 0.05).

**Conclusion:**

DII was positively correlated with blood inflammation indicators, and higher DII and blood inflammation indicators increased the risk of developing cognitive impairment.

## Introduction

With the advent of an aging society, the cognitive decline of the elderly has become a social problem that is important to all humankind and needs to be solved (Hebert et al., 2013; Wang et al., 2021). The number of people living with dementia is increasing worldwide (Afzal et al., 2014). The increasing incidence rates of cognitive impairment and dementia will lead to an increased incidence of various geriatric diseases, which will greatly increase medical investment and impose a heavy socioeconomic burden (Mangialasche et al., 2009; Ruangritchankul et al., 2020). There is now evidence that cognitive impairment is related to human inflammation (Ferrucci and Fabbri, 2018; Irwin and Vitiello, 2019; Barter et al., 2021; Minhas et al., 2021). An adjustable, controlled intake of anti- or proinflammatory foods may be able to modulate the inflammatory state of the body, thereby positively impacting human cognitive function (Aquilani et al., 2020, 2022; van’t Klooster et al., 2020). The foods that people consume in daily life have complex constituents, which make it necessary to explore the impacts of certain combinations of dietary conditions on inflammation and cognitive impairment in humans (Watson et al., 2022). The dietary inflammatory index (DII) is calculated by combining various food components and is a recognized indicator of overall dietary inflammation (Shivappa et al., 2017, 2019; Ryu et al., 2019). There is evidence that DII is negatively correlated with cognitive function (Hayden et al., 2017; Frith et al., 2018; Shin et al., 2018), and studies have also found that DII has no significant effect on cognitive function (Zabetian-Targhi et al., 2021). There is a need to systematically explore the relationship between DII and cognitive function.

The blood inflammation indicators analyzed in the study were collected during physical examinations and recorded in the National Health and Nutrition Examination Survey (NHANES) database. Platelets, platelet–lymphocyte ratio (PLR), and neutrophil–lymphocyte ratio (NLR) have been found to be positively associated with the risks of cerebrovascular and cardiovascular disease (Trakarnwijitr et al., 2017; He et al., 2019). The systemic inflammatory response index [SIRI, calculated as neutrophil count (NE) × (monocyte count)/(lymphocyte count) (Lym)] and the systemic immune-inflammation index [SII, calculated as (peripheral platelet count) × NE/Lym] may be associated with age-related diseases such as those of the cerebrovascular and cardiovascular systems (Jin et al., 2021; Xu et al., 2021). However, there is little comprehensive evidence of the relationships between DII and the white blood cell count (WBC), NE, Lym, NLR, PLR, neutrophil–albumin ratio (NAR), SII, and SIRI, or of their synergistic effects on cognitive function.

We therefore used NHANES data to investigate the relationships of DII and a blood inflammation index with cognition in older Americans, and to explore possible ways to reduce the occurrence of cognitive impairment.

## Materials and methods

### Data source

The data used in our study were derived from the NHANES public database in the United States. All participants provided written informed consent (Wu et al., 2021). There is a dedicated system management system that is responsible for data collection and updates in the NHANES, and the survey data and project information are updated regularly on the website and can be accessed by the public for free (Wu et al., 2021).

### Participants

**FIGURE 1:**
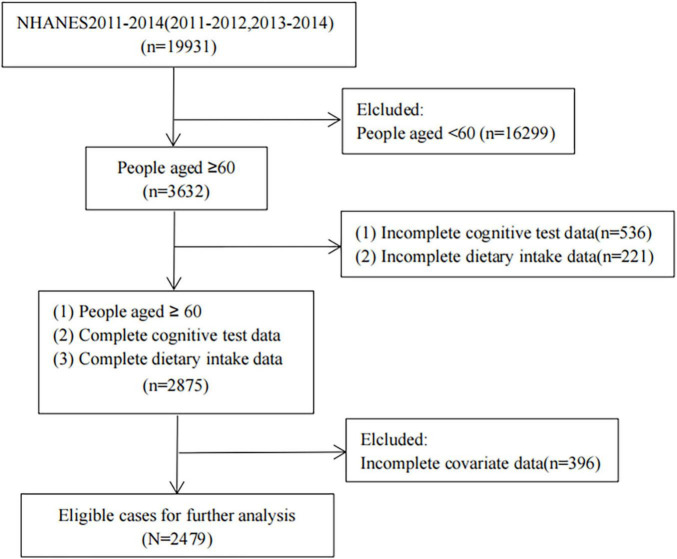
Case inclusion process.

Data on the DII, blood inflammation index, and cognitive performance test scores were obtained from the NHANES for the period from 2011 to 2014 (Yang et al., 2020). All participants or their guardians signed an informed-consent form. We only included people aged 60 years or older, and after further exclusion screening, 2,479 cases were finally included (Figure 1).

### Calculation of DII

This study analyzed 28 of the 45 food components from the original DII: carbohydrates, protein, total fat, alcohol, fiber, cholesterol, saturated fat, MUFA, PUFA, n-3 fatty acids, n-6 fatty acids, niacin, vitamin A, thiamin (vitamin B1), riboflavin (vitamin B2), vitamin B6, vitamin B12, vitamin C, vitamin D, vitamin E, Fe, Mg, zinc, selenium, folic acid, beta-carotene, caffeine, and energy. There is evidence that DII is still useful for predicting overall inflammation when only information on fewer food components is available (Shivappa et al., 2014a). DII calculations were based on a 24-h dietary recall interview or food record of the participant or their guardian (Shivappa et al., 2014b; Wirth et al., 2017). There are standard reference values for each food parameter in the world database. The 24-h dietary recall data were multiplied by standard food parameters from the world database to obtain individual dietary inflammation composite cognitive function scores (Z-scores) relative to the standard global average. We transformed this value into a percentile to reduce bias. Each percentile was doubled, and then 1 was subtracted from it. The percentage values for each food parameter were then multiplied by their respective “overall food parameter-specific inflammatory effect scores” to obtain individual food-specific DII scores. Finally, the DII scores for all individual food components were summed to obtain the “overall DII score” for each person (Shivappa et al., 2014a).

### Cognitive function

The cognitive function assessment consisted of the following four tests: Consortium to Establish a Registry for Alzheimer’s Disease Word Learning (CERAD-WL) test, Animal Fluency (AF) test, Digit Symbol Substitution Test (DSST), and CERAD Delayed Recall (CERAD-DR) test. The CERAD-WL test requires participants to recall as many words as possible after reading ten unrelated words aloud in different orders for a total of 30 points. The CERAD-DR test was administered after the AF test and DSST. Participants were asked to recall words on the CERAD-WL test, which was used to assess transient and delayed learning ability (Rosen, 1983).

The Animal Fluency (AF) tests. Participants were asked to name as many animals as possible within 1 min. The absolute verbal fluency and executive function of the participants were examined (Clark et al., 2009; Sutin et al., 2022).

In the DSST, we asked participants to copy the corresponding symbols into the boxes next to the numbers within 2 min for a total of 133 points (Brody et al., 2019). This test examines the executive function and working memory capacity of the participants.

### Composite cognitive function score

To exclude uneven differences in individual cognitive scores, we used a Z-score consisting of the CERAD-WL test, CERAD-DR test, AF test, and DSST as the total globally standardized cognitive function score. The Z-score was calculated as *Z* = (x-m)/σ, where x is the raw score, m is the overall mean, and σ is the overall SD. A Z-score of<-1 is taken to indicate that the person has cognitive impairment (Wirth et al., 2017; Frith et al., 2018; Zhang et al., 2022).

### Blood inflammation indicators

Data on WBC, NE, Lym, NLR, PLR, NAR, SIRI, SII were extracted from the NHANES database or calculated using extracted peripheral blood counts (Wu et al., 2021).

### Covariates

The possible effects of the following confounders were assessed: age (continuous), sex (male and female), race (Mexican American, other Hispanic, non-Hispanic white persons, non-Hispanic black persons, and non-Hispanic American), marital status (married/living with a partner, widowed/divorced/separated, and unmarried), education level (less than 9th grade, 9–11th grade, high school graduate/GED or equivalent, some college or a degree, and college graduate or above), BMI (continuous), hypertension (yes and no), and diabetes (yes, borderline, and no).

### Statistical analysis

We calculated new sample weights for the data analysis (Liu et al., 2013). If continuous variables did not conform to the normal distribution, they were represented by median (interquartile range) values; otherwise mean (SE) values were used. Regarding intergroup comparisons of baseline data, weighted-sample independent *t*-tests were used for continuous variables, while chi-square tests were used for categorical variables. WBC, NE, Lym, NLR, PLR, NAR, SII, SIRI, and DII were initially considered as continuous variables. In the logistic regression, WBC, NE, Lym, NLR, PLR, NAR, SII, and SIRI were divided into quartile groups (Q1, Q2, Q3, and Q4), and DII was divided into tertile groups (T1, T2, and T3) (Brody et al., 2019). The logistic regression model was adjusted for sex, age, race, marital status, education level, BMI, hypertension, and diabetes. Significant results were indicated by *p* < 0.05. All analyses were performed using R software.

## Results

### General characteristics

The study finally included 2,479 individuals aged ≥60 years. The Z-scores indicated that 426 participants had low cognitive function and 2,053 had normal cognitive function. According to Z-scores, the low-cognitive-function group was older and had higher rates of non-Hispanic black persons, divorced/separated/widowed, lower education levels, hypertension, and diabetes than the normal group (Table 1).

**TABLE 1:** Characteristics of participants [mean (SE)/N (%)].

	Normal cognitive performance (*N* = 2,053)	Low cognitive performance (*N* = 426)	*P*
Age (year)	68.551 (0.205)	73.568 (0.592)	<0.0001**
Gender			1
Male	985 (46.49)	240 (46.50)	
Female	1,068 (53.51)	186 (53.50)	
Race			<0.0001**
Mexican American	161 (2.90)	56 (9.05)	
Non-Hispanic white persons	1,116 (82.46)	131 (54.34)	
Non-Hispanic black persons	420 (6.70)	133 (19.52)	
Other Hispanic	163 (2.57)	85 (13.58)	
Other races	193 (5.37)	21 (3.50)	
Material status (%)			<0.001**
Married/living with partner	1,250 (66.92)	218 (52.36)	
divorced and separated and widowed	657 (27.26)	179 (41.35)	
never married	146 (5.82)	29 (6.28)	
Educational level (%)			<0.0001**
less than 9th grade	106 (3.01)	157 (27.67)	
9–11th grade	239 (8.89)	92 (19.51)	
high school graduate/ged or equivalent	496 (21.78)	90 (25.08)	
some college or a degree	655 (33.33)	56 (17.48)	
college graduate or above	557 (32.98)	31 (10.26)	
Body mass index (%)	29.098 (0.251)	28.382 (0.603)	0.325
Ever told you had high blood pressure (%)			<0.0001**
Yes	1,246 (55.82)	298 (72.16)	
No	807 (44.18)	128 (27.84)	
Doctor told you have diabetes (%)			<0.0001**
Yes	416 (17.23)	147 (32.24)	
Borderline	101 (4.46)	16 (2.96)	
No	1,536 (78.31)	263 (64.81)	
CERAD-WL	20.492 (0.236)	13.757 (0.324)	<0.0001**
CERAD-DR	6.619 (0.110)	3.462 (0.115)	<0.0001**
AF	18.941 (0.173)	11.491 (0.203)	<0.0001**
DSST	55.865 (0.458)	24.456 (0.658)	<0.0001**
The composite cognitive score Z-score	0.549 (0.032)	−1.455 (0.029)	<0.0001**

CERAD-WL, the Consortium to Establish a Registry for Alzheimer’s Disease Word Learning; CERAD-DR, the Consortium to Establish a Registry for Alzheimer’s Disease Delayed Recall; AF, the Animal Fluency. DSST, Digit Symbol Substitution Test. *P < 0.05 and **P < 0.01.

### Comparisons of DII, WBC, NE, Lym, NLR, PLR, NAR, SII, and SIRI between low-cognitive-function group and controls

No differences in LE and PLR were found between the cognitively impaired and normal groups. Patients in the cognitively impaired group had higher DII (*p* < 0.0001), WBC (*p* = 0.004), NE (*p* = 0.003), NLR (*p* = 0.008), NAR (*p* < 0.001), SII (*p* = 0.01), and SIRI (*p* = 0.009) than the normal group (Table 2).

**TABLE 2:** Comparison of dietary inflammatory index and blood inflammatory indicators between the cognitive impairment group and the normal group.

	Total	The composite cognitive score Z-score		
		**Normal cognitive performance**	**Low cognitive performance**	***P*** **-value**
DII	1.353 (0.073)	1.279 (0.079)	2.040 (0.100)	<0.0001**
WBC	6.955 (0.080)	6.906 (0.083)	7.407 (0.156)	0.004**
NE	4.209 (0.051)	4.167 (0.054)	4.606 (0.123)	0.003**
Lym	1.916 (0.033)	1.912 (0.032)	1.953 (0.072)	0.513
NLR	2.502 (0.041)	2.464 (0.039)	2.850 (0.138)	0.008**
PLR	131.252 (2.095)	131.166 (2.146)	132.054 (3.497)	0.79
NAR	0.101 (0.001)	0.095 (0.001)	0.104 (0.002)	<0.001**
SII	561.714 (11.226)	554.512 (11.386)	628.510 (26.768)	0.01*
SIRI	1.440 (0.030)	1.417 (0.031)	1.658 (0.082)	0.009**

DII, dietary inflammatory index; WBC, white blood cell; NE, neutrophil count; Lym, lymphocyte count; NLR, neutrophil–lymphocyte ratio; PLR, platelet-lymphocyte ratio; NAR, neutrophil-albumin ratio; SII, systemic immune inflammation index; SIRI, system inflammation response index. *P < 0.05 and **P < 0.01.

### Correlations of DII, WBC, NE, Lym, NLR, PLR, NAR, SII, and SIRI with the Z-score

Multiple linear regression analysis was performed to analyze the Correlations of DII, WBC, NE, Lym, NLR, PLR, NAR, SII, and SIRI with the Z-score. DII combined with Lym, NLR, PLR, SII, and SIRI, were no correlation with Z-scores after adjusting for all of the abovementioned covariates (*p* > 0.05). However, DII (β = −0.091, *p* < 0.0001) combined with WBC (β = −0.028, *p* = 0.012), NE (β = −0.036, *p* = 0.003), and NAR (β = −1.776, *p* < 0.001) were negatively correlated with Z-scores (Table 3).

**TABLE 3:** Relationship between DII and cognitive function after binding of each blood inflammatory index separately.

	The composite cognitive score Z-score	
	**95% CI**	***P***
**Model 1**		
DII effects	−0.091 (−0.118, −0.065)	<0.0001**
WBC effects	−0.028 (−0.050, −0.007)	0.012*
**Model 2**		
DII effects	−0.092 (−0.118, −0.065)	<0.0001**
NE effects	−0.036 (−0.060, −0.013)	0.003**
**Model 3**		
DII effects	−0.094 (−0.120, −0.068)	<0.0001**
Lym effects	−0.025 (−0.077, 0.028)	0.339
**Model 4**		
DII effects	−0.094 (−0.120, −0.068)	<0.0001**
NLR effects	−0.012 (−0.036, 0.011)	0.284
**Model 5**		
DII effects	−0.094 (−0.120, −0.069)	<0.0001**
PLR effects	0 (0.000, 0.001)	0.268
**Model 6**		
DII effects	−0.091 (−0.118, −0.063)	<0.0001**
NAR effects	−1.776 (−2.732, −0.821)	<0.001**
**Model 7**		
DII effects	−0.094 (−0.120, −0.067)	<0.0001**
SII effects	0 (0.000, 0.000)	0.279
**Model 8**		
DII effects	−0.094 (−0.120, −0.067)	<0.0001**
SIRI effects	−0.019 (−0.053, 0.016)	0.279

Data are all adjusted by age, sex, race, marital status, education level, body mass index, hypertension, diabetes. Models 1–8 show the effects of DII and WBC, NE, Lym, NLR, PLR, NAR, SII, and SIRI on cognitive function, respectively. DII, dietary inflammatory index; WBC, white blood cell; NE, neutrophil count; Lym, lymphocyte count; NLR, neutrophil–lymphocyte ratio; PLR, platelet-lymphocyte ratio; NAR, neutrophil-albumin ratio; SII, systemic immune inflammation index; SIRI, system inflammation response index. *P < 0.05 and **P < 0.01.

### Performance of DII on WBC, NE, Lym, NLR, PLR, NAR, SII, and SIRI in cognitive impairment

The relationship between DII and blood inflammation indices (WBC, NE, Lym, NLR, PLR, NAR, SII, and SIRI) in the cognitively impaired group was further investigated using multiple linear regression. After adjusting for covariates, DII was found to be positively correlated with SII (β = 27.476, *p* = 0.047). No significant associations were found between DII and the other inflammation indicators (*p* > 0.05) (Table 4).

**TABLE 4:** Relationship between DII and each blood inflammatory index in a cognitively impaired population.

	The composite cognitive score Z-score		
	**β**	**95% CI**	***P***
WBC	0.13	(−0.062, 0.321)	0.174
NE	0.092	(−0.031, 0.214)	0.135
Lym	0.027	(−0.034,0.088)	0.370
NLR	0.068	(−0.036, 0.172)	0.188
PLR	2.716	(−1.489, 6.921)	0.195
NAR	0.003	(0.000, 0.006)	0.075
SII	27.476	(0.360, 54.593)	0.047*
SIRI	0.052	(−0.066, 0.171)	0.370

Data are all adjusted by age, sex, race, marital status, education level, body mass index, hypertension, diabetes. DII, dietary inflammatory index; WBC, white blood cell; NE, neutrophil count; Lym, lymphocyte count; NLR, neutrophil–lymphocyte ratio; PLR, platelet-lymphocyte ratio; NAR, neutrophil-albumin ratio; SII, systemic immune inflammation index; SIRI, system inflammation response index. *P < 0.05.

### The role of inflammation indicators in patients with cognitive impairment

A logistic regression approach was used to explore the association between inflammation scores and cognitive impairment risk. Q1 was the reference for all comparisons. First, after adjusting for all the confounding factors that we accounted for, the relationship between inflammatory indicators (DII, WBC, NE, Lym, NLR, PLR, NAR, SII, and SIRI) and the risk of cognitive impairment risk was examined independently (Table 5). The results indicated that T2 and T3 of DII (T2: OR = 1.879, 95% CI = 1.242–2.842; T3: OR = 2.661, 95% CI = 1.745–4.058), Q4 of WBC (OR = 1.778, 95% CI = 1.022–3.096), Q4 of NLR (OR = 1.671, 95% CI = 1.162–2.403), Q4 of NAR (OR = 1.656, 95% CI = 1.094–2.509), Q4 of SII (OR = 1.717, 95% CI = 1.092–2.700), and Q4 of SIRI (OR = 1.563, 95% CI = 1.082–2.258) were risk factors for cognitive impairment. DII combined with WBC, NE, Lym, NLR, PLR, NAR, SII, and SIRI were tested in models 1–8, respectively. Although the combinations of DII and WBC, NE, Lym, and PLR were not found to be significantly associated with the risk of cognitive impairment, higher DII with NLR, NAR, SII, and SIRI significantly increased the risk of cognitive impairment (*p* < 0.05) (Table 6).

**TABLE 5:** The effect of dietary inflammatory index and blood inflammation indicators on cognitive function was analyzed by logistic regression.

	The composite cognitive score Z-score	
	**OR (95% CI)**	***P***
**DII effects**		
T2 (0.74∼2.60) vs. T1 (≤ 0.74)	1.879 (1.242,2.842)	0.005**
T3 (> 2.60) vs. T1 (≤ 0.74)	2.661 (1.745,4.058)	<0.001**
WBC effects		
Q2 (5.60∼6.70) vs. Q1 (≤ 5.60)	1.136 (0.678,1.903)	0.608
Q3 (6.70∼8.10) vs. Q1 (≤ 5.60)	1.299 (0.769,2.196)	0.307
Q4 (> 8.10) vs. Q1 (≤ 5.60)	1.778 (1.022,3.096)	0.043*
**NE effects**		
Q2 (3∼3.90) vs. Q1 (≤ 3)	0.830 (0.481,1.432)	0.481
Q3 (3.90∼5) vs. Q1 (≤ 3)	1.145 (0.726,1.805)	0.539
Q4 (> 5) vs. Q1 (≤ 3)	1.558 (0.912,2.661)	0.099
**Lym effects**		
Q2 (1.50∼1.90) vs. Q1 (≤ 1.50)	0.826 (0.535,1.277)	0.368
Q3 (1.90∼2.30) vs. Q1 (≤ 1.50)	0.639 (0.409,0.997)	0.048*
Q4 (> 2.30) vs. Q1 (≤ 1.50)	1.133 (0.779,1.648)	0.493
**NLR effects**		
Q2 (1.53∼2.07) vs. Q1 (≤ 1.53)	0.969 (0.647,1.451)	0.871
Q3 (2.07∼2.88) vs. Q1 (≤ 1.53)	1.212 (0.788,1.863)	0.359
Q4 (> 2.88) vs. Q1 (≤ 1.53)	1.671 (1.162,2.403)	0.008**
**PLR effects**		
Q2 (92.71∼117.20) vs. Q1 (≤ 92.71)	1.018 (0.744,1.393)	0.903
Q3 (117.20∼150) vs. Q1 (≤ 92.71)	0.799 (0.540,1.181)	0.242
Q4 (> 150) vs. Q1 (≤ 92.71)	1.033 (0.695,1.535)	0.864
**NAR effects**		
Q2 (0.07∼0.09) vs. Q1 (≤ 0.07)	0.853 (0.530,1.372)	0.489
Q3 (0.09∼0.12) vs. Q1 (≤ 0.07)	1.111 (0.754,1.638)	0.575
Q4 (> 0.12) vs. Q1 (≤ 0.07)	1.656 (1.094,2.509)	0.020*
**SII effects**		
Q2 (320∼452.57) vs. Q1 (≤ 320)	1.405 (0.920,2.146)	0.108
Q3 (452.57∼653.05) vs. Q1 (≤ 320)	0.865 (0.543,1.376)	0.518
Q4 (> 653.05) vs. Q1 (≤ 320)	1.717 (1.092,2.700)	0.022*
**SIRI effects**		
Q2 (0.76∼1.13) vs. Q1 (≤ 0.76)	1.334 (0.805,2.209)	0.245
Q3 (1.13∼1.69) vs. Q1 (≤ 0.76)	0.997 (0.665,1.496)	0.988
Q4 (> 1.69) vs. Q1 (≤ 0.76)	1.563 (1.082,2.258)	0.020*

Data are all adjusted by age, sex, race, marital status, education level, body mass index, hypertension, diabetes. DII, dietary inflammatory index; WBC, white blood cell; NE, neutrophil count; Lym, lymphocyte count; NLR, neutrophil–lymphocyte ratio; PLR, platelet-lymphocyte ratio; NAR, neutrophil-albumin ratio; SII, systemic immune inflammation index; SIRI, system inflammation response index. *P < 0.05, **P < 0.01.

**TABLE 6:** Synergistic effects of dietary inflammatory index and blood inflammation indicators on cognitive function.

	The composite cognitive score Z-score	
	**OR (95% CI)**	***P***
**Model 1**		
**DII effects**		
T2 (0.74∼2.60) vs. T1 (≤ 0.74)	1.812 (1.199,2.739)	0.008**
T3 (> 2.60) vs. T1 (≤ 0.74)	2.583 (1.686,3.957)	<0.001**
**WBC effects**		
Q2 (5.60∼6.70) vs. Q1 (≤ 5.60)	1.142 (0.675,1.933)	0.597
Q3 (6.70∼8.10) vs. Q1 (≤ 5.60)	1.301 (0.769,2.201)	0.302
Q4 (> 8.10) vs. Q1 (≤ 5.60)	1.699 (0.970,2.975)	0.062
Model 2	−0.165 (−0.282, −0.048)	0.008*
**DII effects**		
T2 (0.74∼2.60) vs. T1 (≤ 0.74)	1.825 (1.207,2.761)	0.007**
T3 (> 2.60) vs. T1 (≤ 0.74)	2.638 (1.728,4.029)	<0.001**
**NE effects**		
Q2 (3∼3.90) vs. Q1 (≤ 3)	0.789 (0.453,1.375)	0.378
Q3 (3.90∼5) vs. Q1 (≤ 3)	1.111 (0.703,1.754)	0.632
Q4 (> 5) vs. Q1 (≤ 3)	1.478 (0.852,2.564)	0.152
**Model 3**		
**DII effects**		
T2 (0.74∼2.60) vs. T1 (≤ 0.74)	1.920 (1.255,2.937)	0.005**
T3 (> 2.60) vs. T1 (≤ 0.74)	2.678 (1.728,4.152)	<0.001**
**Lym effects**		
Q2 (1.50∼1.90) vs. Q1 (≤ 1.50)	0.792 (0.499,1.256)	0.298
Q3 (1.90∼2.30) vs. Q1 (≤ 1.50)	0.627 (0.390,1.008)	0.053
Q4 (> 2.30) vs. Q1 (≤ 1.50)	1.094 (0.741,1.615)	0.629
**Model 4**		
**DII effects**		
T2 (0.74∼2.60) vs. T1 (≤ 0.74)	1.835 (1.203,2.801)	0.008**
T3 (> 2.60) vs. T1 (≤ 0.74)	2.654 (1.716,4.105)	<0.001**
**NLR effects**		
Q2 (1.53∼2.07) vs. Q1 (≤ 1.53)	0.987 (0.659,1.477)	0.945
Q3 (2.07∼2.88) vs. Q1 (≤ 1.53)	1.198 (0.771,1.861)	0.396
Q4 (> 2.88) vs. Q1 (≤ 1.53)	1.687 (1.176,2.418)	0.007**
**Model 5**		
**DII effects**		
T2 (0.74∼2.60) vs. T1 (≤ 0.74)	1.877 (1.231,2.860)	0.006**
T3 (> 2.60) vs. T1 (≤ 0.74)	2.640 (1.717,4.058)	<0.001**
**PLR effects**		
Q2 (92.71∼117.20) vs. Q1 (≤ 92.71)	1.008 (0.745,1.365)	0.954
Q3 (117.20∼150) vs. Q1 (≤ 92.71)	0.824 (0.557,1.218)	0.307
Q4 (> 150) vs. Q1 (≤ 92.71)	1.046 (0.702,1.558)	0.814
**Model 6**		
**DII effects**		
T2 (0.74∼2.60) vs. T1 (≤ 0.74)	1.815 (1.198,2.749)	0.008**
T3 (> 2.60) vs. T1 (≤ 0.74)	2.610 (1.712,3.980)	<0.001**
**NAR effects**		
Q2 (0.07∼0.09) vs. Q1 (≤ 0.07)	0.830 (0.509,1.354)	0.430
Q3 (0.09∼0.12) vs. Q1 (≤ 0.07)	1.056 (0.712,1.564)	0.773
Q4 (> 0.12) vs. Q1 (≤ 0.07)	1.577 (1.045,2.379)	0.032*
**Model 7**		
**DII effects**		
T2 (0.74∼2.60) vs. T1 (≤ 0.74)	1.639 (0.995,2.702)	0.052
T3 (> 2.60) vs. T1 (≤ 0.74)	2.140 (1.325,3.458)	0.005**
**SII effects**		
Q2 (320∼452.57) vs. Q1 (≤ 320)	1.513 (0.929,2.464)	0.089
Q3 (452.57∼653.05) vs. Q1 (≤ 320)	0.944 (0.589,1.513)	0.794
Q4 (> 653.05) vs. Q1 (≤ 320)	1.996 (1.251,3.183)	0.007**
**Model 8**		
**DII effects**		
T2 (0.74∼2.60) vs. T1 (≤ 0.74)	1.883 (1.243,2.851)	0.005**
T3 (> 2.60) vs. T1 (≤ 0.74)	2.627 (1.727,3.997)	<0.001**
**SIRI effects**		
Q2 (0.76∼1.13) vs. Q1 (≤ 0.76)	1.371 (0.879,2.138)	0.151
Q3 (1.13∼1.69) vs. Q1 (≤ 0.76)	0.853 (0.529,1.378)	0.491
Q4 (> 1.69) vs. Q1 (≤ 0.76)	1.679 (1.052,2.680)	0.032*

Data are all adjusted by age, sex, race, marital status, education level, body mass index, hypertension, diabetes. DII, dietary inflammatory index; WBC, white blood cell; NE, neutrophil count; Lym, lymphocyte count; NLR, neutrophil–lymphocyte ratio; PLR, platelet-lymphocyte ratio; NAR, neutrophil-albumin ratio; SII, systemic immune inflammation index; SIRI, system inflammation response index. *P < 0.05 and **P < 0.01.

## Discussion

We systematically explored the relationships between dietary inflammation, blood inflammation indicators, and cognitive impairment. Our study found that DII combined with WBC, NE, NLR, NAR, SII, and SIRI were considerably higher in the low-cognitive-ability group than in the normal group. DII, WBC, NE, NLR, NAR, SII, and SIRI were negatively correlated with Z-scores. DII combined with WBC, NE, and NAR were all negatively correlated with Z-scores. DII was positively correlated with blood inflammation indicators. Older adults with higher levels of DII and blood inflammation indicators (NLR, NAR, DII, and SIRI) were at a higher risk of cognitive impairment.

In the elderly, the body becomes less functional and more susceptible to inflammation (Tangestani Fard and Stough, 2019). Human inflammation indicators (NLR, PLR, NAR, SII, and SIRI) have been indicated to be potentially related to various health hazards in the elderly, including cardiovascular and cerebrovascular diseases (Trakarnwijitr et al., 2017; He et al., 2019; Dong et al., 2020; Jin et al., 2021; Li et al., 2021; Xu et al., 2021). Platelets, PLR, NLR, and NAR are associated with the risks of stroke and cardiovascular disease (Trakarnwijitr et al., 2017; He et al., 2019). Our results found that DII combined with WBC, NE, NLR, NAR, SII, and SIRI were considerably higher in the cognitive-impairment group than in the normal group. DII, WBC, NE, Lym and NAR were negatively correlated with Z-scores, which was similar to the results of previous studies (Xu et al., 2021).

DII represents the combined inflammatory profile of the human diet, and the relationship between DII and many risk factors has been demonstrated for age-related diseases (Frith et al., 2018). Our study found that DII was significantly associated with SII. Although no significant correlation was found with other blood inflammation indicators, SII is a more-reliable and representative marker of inflammation, so we believe that the evidence here is sufficient and convincing. However, our conclusion differed from those of previous studies, including that of Wang et al. (2022), who found that DII was significantly correlated with SIRI but not with SII. The possible reasons for this are that the previous study involved Chinese subjects, and Chinese and American diets are very different, there are various racial differences, and the Montreal Cognitive Assessment scale differs from the Z-score calculated by our CERAD-WL, CERAD-DR, AF, and DSST. However, both studies suggested that DII contributes to chronic inflammation development in humans.

The correlation between DII and cognitive impairment has been previously explored in different regions. Hayden et al. (2017) found that DII scores were positively associated with the risk of developing cognitive impairment, Shin et al. (2018) found that higher DII indicated higher cognitive impairment risk, and Frith et al. (2018) also found that higher DII scores were negatively associated with cognitive impairment risk. Our study found that DII, WBC, NE, NLR, NAR, SII, and SIRI were all negatively associated with Z-scores. DII combined with WBC, NE, and NAR were negatively correlated with Z-scores. This suggests that DII and blood inflammation indices can synergistically serve to affect cognitive function.

Logistic regression was used to further investigate the synergistic effect of blood inflammation index and DII on cognitive impairment risk. The results indicated that older adults with higher DII and levels of blood inflammation indicators (NLR, NAR, DII, and SIRI) were at a higher risk of cognitive impairment, which was similar to the results of previous studies (Jin et al., 2021). The possible mechanism is that inflammation indicators can cross the blood–brain barrier to inflame nerves, leading to neurodegeneration (d’Avila et al., 2018; Godos et al., 2020; Leng and Edison, 2021). A comprehensive assessment of diet and blood inflammation can help us take early steps to develop a rational dietary intervention plan and protect cognitive function.

This study had some limitations. First, because the study had a cross-sectional design, it was not possible to infer causal relationships between dietary and blood inflammatory indicators, and cognition. Second, dietary inflammatory indicators calculated from dietary intake data obtained from 24-h dietary recall might not accurately reflect individual dietary intakes and are subjected to recall bias. Third, we did not identify the cause of any impairment, such as Alzheimer’s disease, Lewy-body dementia, or vascular dementia.

Our study also shows strengths and important originality. First of all, the study has a rich sample size and is analyzed only in older adults over 60 years of age, which has a strong social significance. In addition, the study used relatively accurate dietary data. Finally, the cognitive impairment composite -z score was created by summing the z scores [(individual test score - mean score)/SD] of these three individual tests (DSST, AFT, CERAD), with good sensitivity and avoiding ceiling and floor effects. For cognitive purposes our findings emphasize the importance of an anti-inflammatory diet with clinical implications.

In conclusion, we found that dietary and blood inflammation indicators were negatively associated with cognitive function in an elderly American population, and that dietary inflammation indicators were also negatively associated with cognitive function when combined with blood inflammation indicators. DII was positively correlated with blood inflammation. Older adults with higher DII and blood inflammation indicator levels were at a higher risk of cognitive impairment. An ideal dietary intake among older adults was associated with improved cognitive function, and future studies should therefore further investigate the interrelationships and the mechanisms underlying their effects on cognition.

## Data availability statement

Publicly available datasets were analyzed in this study. This data can be found here: https://www.cdc.gov/nchs/nhanes/index.htm.

## Ethics statement

The data used in our study were derived from the NHANES public database in the United States. All participants provided written informed consent. The patients/participants provided their written informed consent to participate in this study.

## Author contributions

WL, SL, and WZ: conceptualization. WL: methodology and data curation. YS: software. WL, SL, and GY: validation. WL, WZ, and ZC: writing – original draft preparation. WL and WZ: writing – review and editing. JL: visualization. All authors have read and agreed to the published version of the manuscript.
